# Deformation-Induced Electromagnetic Reconfigurable Square Ring Kirigami Metasurfaces

**DOI:** 10.3390/mi15121493

**Published:** 2024-12-13

**Authors:** Xuanqing Fan, Zijian Pan, Yunfan Zhu, Min Li, Yunpeng Ma, Yuhang Li

**Affiliations:** 1 Tianmushan Laboratory, Yuhang District, Hangzhou 311115, China; 2 Hangzhou International Innovation Institute, Beihang University, Hangzhou 311115, China; limin@buaa.edu.cn; 3 School of Aeronautic Science and Engineering, Beihang University, Beijing 100191, China

**Keywords:** flexible frequency selective surface, krigami, metasurface, mechanical deformation tuning

## Abstract

The continuous expansion of wireless communication application scenarios demands the active tuning of electromagnetic (EM) metamaterials, which is essential for their flexible adaptation to complex EM environments. However, EM reconfigurable systems based on intricate designs and smart materials often exhibit limited flexibility and incur high manufacturing costs. Inspired by mechanical metastructures capable of switching between multistable configurations under repeated deformation, we propose a planar kirigami frequency selective surface (FSS) that enables mechanical control of its resonant frequency. This FSS is composed of periodically arranged copper square-ring resonators embedded in a kirigami-structured ecoflex substrate. Through simple tensile deformation, the shapes and positions of the square-ring resonators on the kirigami substrate are altered, resulting in changes to the coupling between capacitance and inductance, thereby achieving active tuning. Combining EM finite element simulations and transmittance measurements, we demonstrate that biaxial mechanical stretching allows for continuous adjustment of the FSS resonant frequency and −10 dB bandwidth. Additionally, the FSS exhibits excellent polarization and incident angle stability. Structural parameterization of the square-ring kirigami FSS was conducted to elucidate the deformation–electromagnetic coupling mechanism underlying the active tuning. These insights provide a foundation for guiding the application of square-ring kirigami FSS in various practical engineering domains.

## 1. Introduction

Metasurfaces, composed of artificially fabricated sub-wavelength-thick structures arranged in a periodic and infinite manner [1,2], leverage the interactions between light, material, and structure to achieve specific functionalities. They have found extensive applications in electromagnetics, such as artificial magnetic conductors [3,4,5], and EM bandgap surfaces [6]. Notably, frequency selective surfaces (FSSs), a specialized category of these structures [7], play a pivotal role in controlling fundamental EM wave transmission properties. Researchers have identified intriguing functionalities of FSS metasurfaces, including polarization manipulation [8,9,10], phase modulation [11], absorption [12,13,14], and abnormal reflection [15,16]. These capabilities have propelled their adoption in diverse applications such as radar radomes [17], stealth absorbers [18], and polarizers [19], garnering significant interest in the scientific community.

With the rapid expansion of wireless communication and the increasing diversification of wireless services, the demand for flexible, adaptive, and reconfigurable EM systems has grown substantially. However, conventional FSS structures are limited by fixed operational frequencies and bandwidths once designed and fabricated. With the growing complexity of EM environments and diverse application scenarios, there is an urgent need for FSSs capable of dynamically tuning their EM transmission properties. This adaptability could extend operational frequency ranges, facilitate functional switching, and address the requirements of various EM applications [20].

Reconfigurable FSS designs typically fall into two categories: EM tuning devices and switching-based devices. EM tuning methods depend on external stimuli, such as magnetic fields [21,22], temperature variations [23,24], light [25], or voltage [26], to modulate the EM properties of functional materials. Switching-based methods, by contrast, employ microelectromechanical (MEMS) switches [27] or varactor diodes [28] to modify the current distribution within FSS unit cells. However, the biasing and control circuits necessary for active components increase system complexity, introduce conductive losses, and may generate interference and reflection, thereby degrading overall performance, particularly at high frequencies [29].

The rapid development of flexible, easily fabricated, and high-sensitivity metasurfaces has opened new pathways for manipulating EM waves through mechanical methods [30]. Yao et al. [31,32] introduced bound states in the continuum into the design of terahertz flexible metasurfaces, enabling high-Q sensitivity detection of micro-curvatures. Lin et al. [33] developed a parabolic metallic element metasurface based on a deformable flexible substrate, achieving a tunable resonance frequency range through mechanical modulation. Additionally, Fan et al. [34,35] quantitatively tuned the resonance frequency and bandwidth by employing out-of-plane buckling of metallic surfaces. Recent advances in manufacturing technologies have expanded the concept of kirigami deformation [36,37] into the realm of EM metasurfaces. Kirigami structures, known for their large deformation amplitudes and diverse morphologies, introduce new design freedoms to conventional FSSs. Han et al. [38] investigated a mechanically reconfigurable 3D chiral metamaterial produced through the compression of an origami-inspired structure on a prestrained substrate, enabling active circular dichroism modulation. Xing et al. [39] combined double split-ring resonators with nano-kirigami designs to achieve broadband, high-efficiency linear polarization switching in the near-infrared spectrum.

This study introduces a novel kirigami-inspired frequency-selective metasurface with reconfigurable resonance frequency and bandwidth. The metasurface consists of square-ring resonators attached to a flexible kirigami substrate. Simple biaxial stretching induces in-plane rotation of the resonators, modifying the capacitance-inductance coupling between them and enabling active tuning of the resonance frequency and bandwidth. Mechanical and EM experiments and simulations were performed to characterize the metasurface’s stretch-induced deformation and its frequency-selective tuning properties. A 40% biaxial strain increased the resonance frequency by 0.8 GHz and expanded the −10 dB bandwidth by 1.73 GHz, resulting in a 7.8% frequency shift. EM experiments also demonstrated the metasurface’s stability across incident angles, highlighting its potential for complex EM device applications.

## 2. Results and Discussion

**Figure 1:**
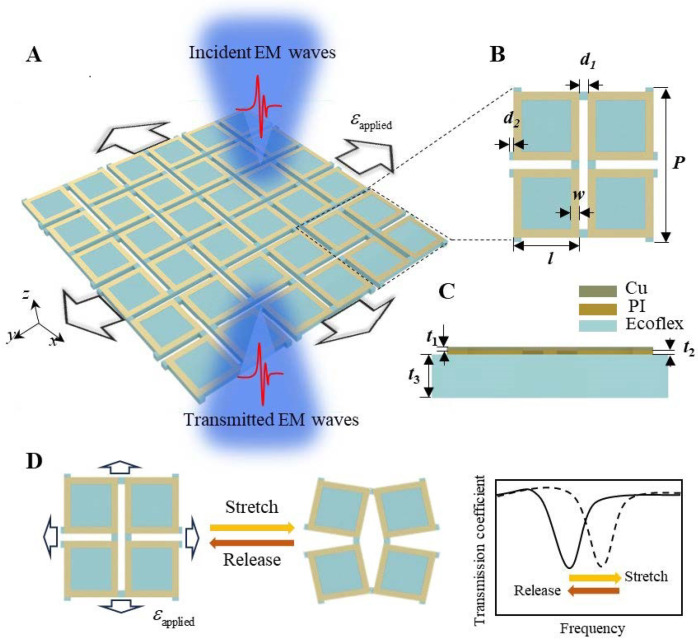
(**A**) Schematic of the square-ring kirigami FSS with altered EM wave transmission characteristics under biaxial tensile strain; (**B**) geometric dimensions and (**C**) cross-sectional view of the unit cell; (**D**) process and mechanism of resonant frequency tuning of the square-ring kirigami FSS through mechanical deformation.

The square-ring kirigami FSS metasurface consists of a periodically cut flexible silicone substrate ecoflex with periodically arranged metallic copper square-ring resonators adhered to its surface. The fabrication process of the square-ring kirigami FSS is detailed in the Section 4. EM waves are selectively transmitted through the metasurface along the negative *z*-axis. The unit cell of the metasurface, depicted in Figure 1B, comprises two layers distinguished by their components and shapes, with a cross-sectional schematic shown in Figure 1C. The top layer consists of four square rings made of stacked Cu and PI layers, while the bottom kirigami ecoflex structure features four squares connected by hinges. The geometric parameters are as follows: unit cell period *P* = 18 mm, square ring side length *l* = 8 mm, ring width *w* = 1 mm, kirigami hinge dimensions *d*_1_ = 1 mm, and *d*_2_ = 0.5 mm. The thicknesses are *t*_1_ = 0.018 mm for Cu, *t*_2_ = 0.0225 mm for PI, and *t*_3_ = 1 mm for ecoflex. When subjected to biaxial tensile deformation, the arrangement of the metallic square-ring resonator undergoes significant changes, leading to variations in the EM transmission coefficient, as illustrated in Figure 1D. The resonant frequency exhibits a desirable shift in response to applied and released strain, embodying the design principle of the square-ring kirigami FSS metasurface introduced in this study.

**Figure 2:**
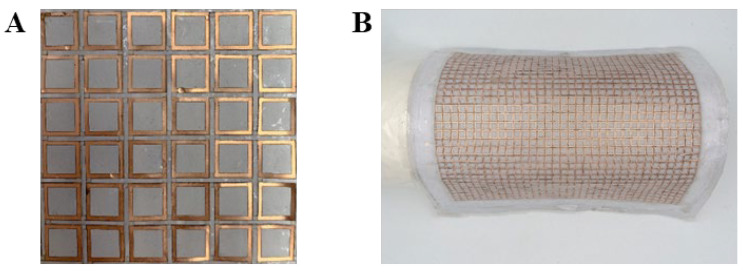
(**A**) Optical photographs of the flat square-ring kirigami FSS; (**B**) optical photograph of the square-ring kirigami FSS attached to the surface of a cylinder.

By utilizing a flexible and stretchable kirigami-patterned substrate, the tensile strain is primarily concentrated at the substrate hinges, leaving the metallic square rings nearly strain-free. This design enables the FSS to withstand significant biaxial tensile strain, facilitating its conformal attachment to curved surfaces. Figure 2A,B present optical images of the square-ring kirigami FSS in its flat and bent configurations, respectively. The structure can adapt to complex cylindrical surfaces through bending and stretching conformally.

When the square-ring kirigami FSS is tightly attached to non-developable curved surfaces, mechanical tensile deformation inevitably occurs. To evaluate the impact of such deformation on its EM transmission performance, it is essential to investigate its mechanical deformation mechanism. The deformation behavior of the square-ring kirigami FSS under biaxial tensile strain was studied using the mechanical simulation software ABAQUS 2022 and a biaxial tensile tester. The processes for mechanical simulation and measurement are detailed in the Methods section.

**Figure 3:**
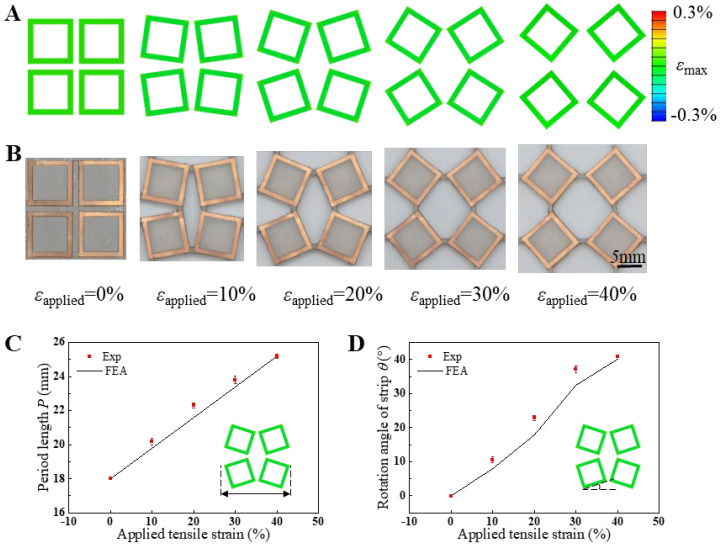
The 2D planar morphology of the composite layer and maximum principal strain (scale bar: 5 mm) under biaxial tensile strains of 0%, 10%, 20%, 30%, and 40%, obtained from (**A**) simulation and (**B**) experimental measurements; the variation of (**C**) unit cell period length and (**D**) rotation angle of the copper strip via different biaxial tensile strains.

Figure 3A,B depict the finite element analysis (FEA) results and experimental observations of mechanical deformation in the square-ring kirigami FSS under biaxial tensile strains of 0%, 10%, 20%, 30%, and 40%, respectively. The deformation patterns observed in the simulations align closely with those in the experimental optical images throughout the tensile deformation range from 0% to 40%. With increasing applied strain, the periodic length of the unit cells in the square-ring kirigami FSS expands gradually, accompanied by an increasing rotation angle of the Cu square rings, while maintaining symmetry along the x- and y-axes. The unique mechanical properties of the kirigami structure ensure that strain during stretching is primarily concentrated in the hinge regions connecting adjacent square units on the ecoflex substrate. In contrast, the interior regions of the square units remain almost strain-free. Consequently, the Cu square rings situated within these regions can be regarded as strain-free zones. Correspondingly, the FEA results also reveal that the maximum principal strain in the Cu square rings is negligible, significantly below the copper yield strain of 0.3% [40]. On the other hand, the ecoflex substrate exhibits excellent fatigue resistance under relatively low tensile strain conditions (less than 40%) due to its hyperelastic mechanical properties [41,42,43]. This feature underpins the mechanical durability of the square-ring kirigami FSS, allowing it to endure repeated tensile deformation without compromising its service life. It is noteworthy that biaxial tensile deformation induces purely in-plane deformation in the square-ring kirigami FSS. This attribute substantially addresses the limitations of three-dimensional buckled FSS structures, which are prone to instability under similar conditions. The resulting improvement underscores the mechanical robustness and versatility of this metasurface design.

To quantitatively analyze the impact of biaxial tensile strain on the deformation morphology of the Cu components, Figure 3C presents the variation in unit cell periodicity with increasing biaxial tensile strain from 0% to 40%, as observed through both simulations and experiments. Similarly, Figure 3D illustrates the relationship between the rotation angle of the Cu square rings and the applied biaxial tensile strain. The unit cell periodicity exhibits a linear increase with biaxial tensile strain, attributable to the significantly lower stiffness of the Cu/PI composite layer compared to the ecoflex substrate. Consequently, the deformation in the x-y plane is predominantly governed by the substrate. A strong agreement is observed between experimental data and simulation results. The rotation angle of the Cu/PI composite layer does not increase linearly with tensile strain. Notably, as the strain approaches 30%, the rotation angle begins to plateau, eventually reaching a maximum of approximately 41°. This behavior arises from the unique mechanical properties of the kirigami structure, which limit the rotation angle. Beyond this point, further increases in tensile strain do not induce additional rotation of the Cu components. Instead, the strain is accommodated by increased stretching at the hinge regions of the ecoflex substrate, where adjacent squares are interconnected.

**Figure 4:**
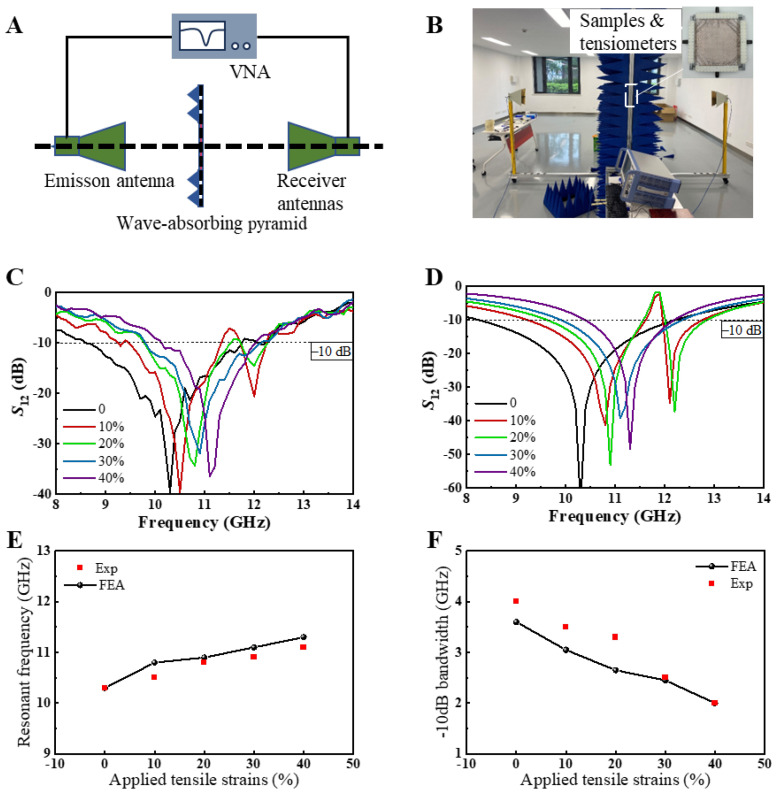
(**A**) Schematic of the experimental setup for measuring the transmittance of the sample using the free-space method; (**B**) photograph of the setup with the sample mounted on the tension meters. The EM wave transmittance coefficient *S*_12_ of the square-ring kirigami FSS under biaxial tensile strains of 0%, 10%, 20%, 30%, and 40% obtained from (**C**) experimental measurements and (**D**) simulation; (**E**) resonant frequency and (**F**) −10 dB bandwidth variation of the square-ring kirigami FSS via biaxial tensile strains.

To investigate the frequency-selective properties of the square-ring kirigami FSS, we employed a free-space EM measurement method to evaluate its transmission characteristics for EM waves. Figure 4A,B depict the schematic and physical setup of the experimental apparatus, respectively. The inset in Figure 4B highlights the sample and tensile device used in the measurements. The sample dimensions were 250 mm × 250 mm, significantly larger than the area exposed to the transmitted electromagnetic wave radiation. According to Saint-Venant’s Principle, the central region of the sample would undergo uniform stretching as the boundary effects caused by the tensile apparatus dissipate rapidly. During electromagnetic measurements, the temperature was maintained at 23–25 °C, and the humidity at 40–50% through controlled air conditioning, minimizing the impact of environmental factors on the stability of the EM sample. Detailed descriptions of the free-space EM measurement procedure are provided in the Methods section. Figure 4C,D present the transmission coefficients of the square-ring kirigami FSS within the 8–14 GHz range under biaxial tensile strains of 0%, 10%, 20%, 30%, and 40%, as obtained from both experimental measurements and simulations. The results demonstrate excellent agreement between the experimental and simulated data, underscoring the stable band-stop frequency-selective properties of the kirigami metasurface and the reliability of its strain-controlled EM performance. Notably, when the biaxial tensile strain is 0%, the square-ring kirigami FSS exhibits a typical single-band band-stop frequency-selective response. As the strain increases to 10% and 20%, the structure transitions to a dual-band band-stop response. With further strain increases to 30% and 40%, the single-band response reemerges. This behavior is primarily attributable to the intrinsic properties of the Cu square rings, which constitute the fundamental unit cell of a single-band band-stop metasurface. Under low biaxial tensile strain, the rotation of the Cu square rings disrupts the symmetry of the metallic unit cells along the x- and y-axes, as shown in Figure 3A,B, leading to the dual-band response. At higher tensile strains, the continued rotation of the Cu square rings restores the structural symmetry along the x- and y-axes, approximating the configuration of a single Cu square ring, thereby reverting to the single-band response. The slight irregularities observed in the experimental transmission curves can be attributed to the limited power output of the horn antennas and the precision constraints of the experimental apparatus.

To further examine the impact of biaxial tensile strain on the frequency-selective properties of the square-ring kirigami FSS, Figure 4E,F illustrate the variation in resonance frequency and −10 dB bandwidth with increasing strain. The dashed line in the figures represents the −10 dB reference level, intersecting the experimental and simulation curves at two points. The frequency difference between these intersection points corresponds to the −10 dB bandwidth. Both experimental and simulation results consistently show that as the square-ring kirigami FSS undergoes biaxial tensile strain, the resonance frequency increases from 10.3 GHz to 11.1 GHz, resulting in a shift of 0.8 GHz. Simultaneously, the −10 dB bandwidth decreases from 3.62 GHz to 1.89 GHz, reflecting a reduction of 1.73 GHz. These two parameters are commonly employed to evaluate the EM performance of FSS. The resonance frequency shift rate is calculated to be 7.8%, while the −10 dB bandwidth variation rate reaches 47.7%, demonstrating a pronounced EM tuning effect.

Flexible FSSs attached to non-developable curved surfaces are subjected not only to tensile deformation but also to a certain degree of bending. This bending causes EM waves to impinge on the FSS at an oblique angle rather than perpendicularly. Furthermore, in practical applications, precisely controlling the angle of incidence of EM waves on the FSS is often unrealistic. Therefore, to account for the practical application scenarios of the square-ring kirigami FSS, it is crucial to investigate the stability of its EM wave transmission performance under varying angles of incidence.

**Figure 5:**
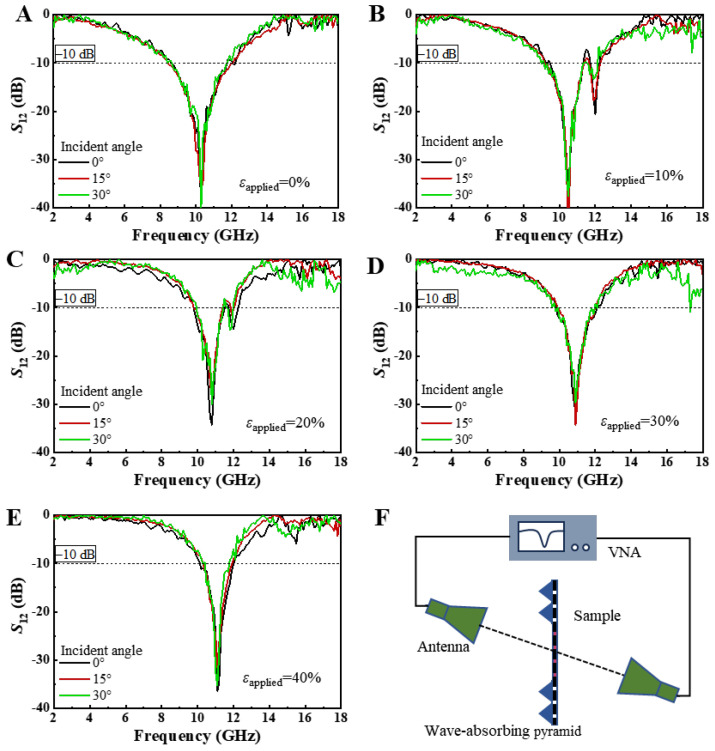
The EM wave transmittance coefficient *S*_12_ at different incident angles (0° to 30°) of the square-ring kirigami FSS under biaxial tensile strains of (**A**) 0%, (**B**) 10%, (**C**) 20%, (**D**) 30%, and (**E**) 40% obtained through free-space EM experiments; (**F**) schematic of the experimental setup for oblique-incidence EM wave measurements.

Figure 5A–E display the transmission coefficients of EM waves incident at angles ranging from 0° to 30° on the square-ring kirigami FSS, which is subjected to biaxial tensile strains of 0%, 10%, 20%, 30%, and 40%, as measured by the free-space EM method. Figure 5F presents the schematic of the experimental setup for measuring the transmission under oblique incidence. The angle between the axis of the horn antenna and the normal to the sample plane is defined as the incident angle. It is evident that regardless of the strain applied to the sample, varying the angle of incidence does not result in significant changes in the transmission performance of the square-ring kirigami FSS. For instance, when the biaxial tensile strain is 40%, as shown in Figure 5E, the change in incidence angle causes a frequency shift of less than 0.1 GHz (or 0.9%), indicating that the FSS exhibits stable EM response under different angles of incidence. Two-dimensional planar structures are often sensitive to variations in the angle of incidence. However, the square-ring kirigami FSS shows a notable advantage in this regard. Therefore, when it is closely adhered to a complex non-developable surface, where oblique angles inevitably arise, its transmission performance remains highly stable.

As previously discussed, the planar square-ring kirigami FSS can actively modulate its EM wave characteristics through biaxial tensile deformation. However, in practical EM applications, there are diverse requirements for wave modulation. Therefore, a parametric structural analysis can be employed to investigate the tuning effects of the planar square-ring kirigami FSS under biaxial tensile strain with varying dimensions. This approach will facilitate the application of this structure across a broader range of fields. The following section explores the influence of periodicity and ring width on the EM wave tuning properties of the planar square-ring kirigami FSS.

**Figure 6:**
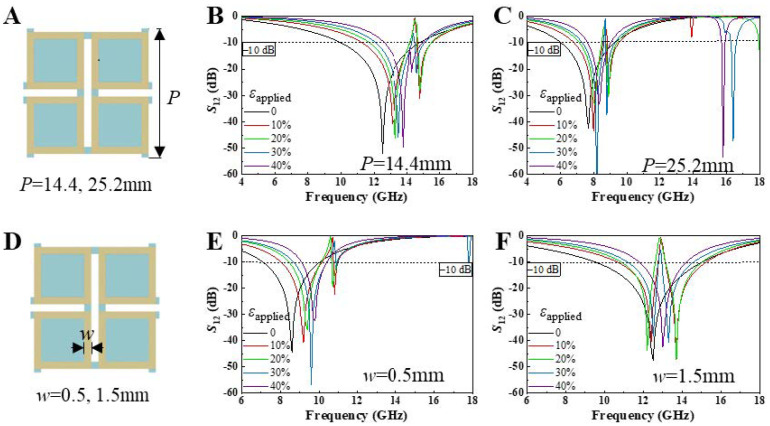
(**A**) Unit cell period length *P* of the square-ring kirigami FSS; the effect of 40% biaxial tensile strain on the EM wave transmittance when *P* is (**B**) 14.4 mm and (**C**) 25.2 mm; (**D**) square-ring width *w* of the square-ring kirigami FSS; the effect of 40% biaxial tensile strain on the EM wave transmittance when *w* is (**E**) 0.5 mm and (**F**) 1.5 mm.

Figure 6A–C investigate the influence of periodicity on the tuning effects of the planar square-ring kirigami FSS. Figure 6B,C show the variation in EM transmission with a periodicity of 14.4 mm and 25.2 mm, respectively, under 40% biaxial tensile strain. Both metasurfaces exhibit excellent frequency-selective properties, with the resonance frequency monotonically increasing as biaxial tensile strain is applied. For the periodicity of 14.4 mm, the resonance frequency increases from 11.4 GHz to 13.8 GHz, while the −10 dB bandwidth decreases from 4.85 GHz to 2.53 GHz. For the periodicity of 25.2 mm, the resonance frequency increases from 7.7 GHz to 8.3 GHz, and the −10 dB bandwidth decreases by 1.82 GHz. As the periodicity increases, the resonance frequencies at all strain states shift toward lower frequencies. Moreover, it is evident that smaller-period metasurfaces exhibit higher electromagnetic tuning sensitivity.

Figure 6D–F illustrate the EM tuning effects of the planar square-ring kirigami FSS with square-ring widths of 0.5 mm and 1.5 mm, respectively, under 40% biaxial tensile strain. Both metasurfaces exhibit excellent frequency-selective properties, with the resonance frequency monotonically increasing as biaxial tensile strain is applied. As the square-ring width increases, the resonance frequencies at all strain states shift toward higher frequencies. Specifically, when the square-ring width is 0.5 mm, the resonance frequency increases from 8.6 GHz to 9.8 GHz, while the −10 dB bandwidth decreases by 1.64 GHz; when the square-ring width is 1.5 mm, the resonance frequency increases from 12.2 GHz to 13 GHz, and the −10 dB bandwidth decreases by 3.08 GHz. From the bandwidth perspective, it is evident that metasurfaces with larger square-ring widths offer a distinct advantage in mechanical tuning.

**Figure 7:**
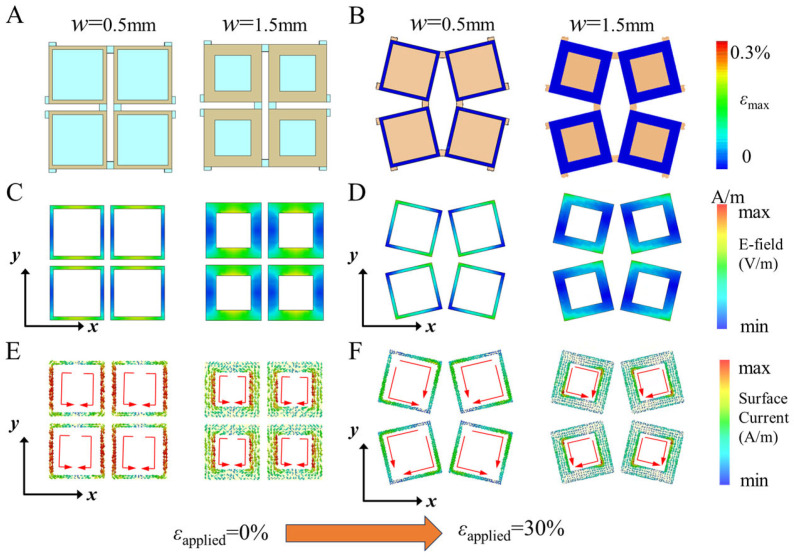
(**A**) the simulation model and (**B**) the strain distribution of the square-ring kirigami FSS with ring widths *w* of 0.5 mm and 1.5 mm when the 30% tensile strain is applied; the metal surface electric field distribution (**C**) before and (**D**) after deformation; the surface current distribution of the metal surface (**E**) before and (**F**) after deformation.

We observed that increasing the period length of the metasurface results in a significant shift of the resonance frequency to lower values, as larger-period metasurfaces resonate more effectively with EM waves of longer wavelengths. As illustrated in Figure 7, we aim to explore the tuning mechanism more comprehensively by examining the impact of square-ring width variation on both the mechanical and electromagnetic properties of the metasurface. Figure 7B illustrates the strain distribution of the FSS metal square rings under 30% tensile strain, as determined through mechanical simulations. It is evident that, regardless of the square ring width, the strain in the metal structure remains significantly below the fracture strain threshold of 0.3%, which is in agreement with prior findings. Additionally, the deformation is observed to be confined within the plane. Figure 7C–F show the electric field and surface current distributions before and after deformation for square ring widths of 0.5 mm and 1.5 mm. Prior to deformation, both the electric field and surface current are mainly concentrated along the walls of the square ring. After deformation, however, these distributions shift and concentrate at the corners of the square ring, exhibiting a distinct asymmetry. This asymmetry leads to the coupling of EM transmission modes [32], which explains the previously observed dual-band frequency-selective blocking phenomenon.

## 3. Conclusions

Inspired by traditional kirigami structures, this study designs and fabricates a planar square-ring kirigami FSS that allows for the mechanical biaxial tensile control of EM wave transmission characteristics. High-performance metallic square-ring unit cells are periodically attached to a flexible kirigami structure substrate. EM wave transmission characteristics, such as resonance frequency and −10 dB bandwidth, can be continuously tuned via mechanical biaxial stretching deformation. The planar square-ring kirigami FSS is expected to substantially overcome the limitations of three-dimensional buckling FSS structures, as it undergoes exclusively in-plane deformation under mechanical biaxial tensile loading. The Cu square rings rotate by a certain angle, altering the shape and dimensions of the unit cell. This change allows for active, continuous tuning of the resonance frequency and −10 dB bandwidth when exposed to EM waves. The results from both simulations and experiments are in good agreement, demonstrating that the planar square-ring kirigami FSS exhibits band-stop EM wave frequency-selective characteristics. In the initial state, with 0% biaxial strain, the resonance frequency is 10.3 GHz with a −10 dB bandwidth of 3.9 GHz. Applying 40% biaxial tensile strain increases the resonance frequency by 0.8 GHz and the −10 dB bandwidth by 1.73 GHz, yielding a resonance frequency variation rate of 7.8%. Additionally, experimental measurements indicate that the planar square-ring kirigami FSS exhibits excellent angular stability for incident angles ranging from 0° to 30°. Square-ring FSSs have significant potential for practical applications, particularly in fields such as intelligent communication, radar detection, and interference protection in complex electromagnetic environments. However, challenges related to environmental adaptability, dynamic response speed, and manufacturing integration need to be addressed through material innovation, integration of AI with sensor networks, and optimization of manufacturing processes. In the future, with advancements in technology, the multifunctionality and adaptive capabilities of square-ring chiral FSSs will be further enhanced, laying the foundation for their broader engineering applications.

## 4. Method

The fabrication process of the square-ring kirigami FSS is described as follows. First, the square-ring metal resonators are prepared. A commercial PI copper-clad film is cut into the designed periodic square-ring shapes using a programmable ultraviolet laser cutter. Next, the metal resonators are picked up. To maintain the periodicity of the square-ring unit cells, water-soluble tape (WST, AQUASOL, Shanghai, China) is used to simultaneously pick up the periodically arranged metal unit cells. The ecoflex substrate is then prepared. ecoflex 00-30 (Smooth-On, Beijing, China) components A and B are mixed in a 1:1 ratio, thoroughly blended using a deaerating stirrer to eliminate bubbles, and poured into an acrylic mold for curing. Commercial adhesive (Dinglifeng, Taizhou, China) is applied to the surface of the periodic square-ring unit cells fixed with water-soluble tape. The metal unit cells are bonded to the ecoflex substrate, and deionized water is used to wash away the water-soluble tape. Finally, the ecoflex substrate is cut. A programmable ultraviolet laser cutter is used to cut the ecoflex bonded with the metal unit cells into the desired kirigami shape, resulting in the fabrication of a 306 mm × 306 mm square-ring kirigami FSS.

The process of mechanical measurement is described as follows. A biaxial tensile device, assembled from a sliding stage (Hongyuantai, Qingdao, China) and 3D-printed clamps, is used to stretch the sample equidistantly at a preset strain rate, as shown in the inset of Figure 4B. An optical microscope is employed to capture the 2D surface morphology of the square-ring kirigami FSS without contacting the sample. Image data are processed using a custom program developed in commercial mathematical software, Matlab 2021, which extracts coordinate information of the selected points. This allows for the measurement of the periodic length and the rotation angle of the Cu strips.

The mechanical simulation process is described as follows. Mechanical simulations were conducted using the commercial software ABAQUS 2022 to model the tensile deformation process of the sample, sequentially obtaining the post-deformation configurations. The maximum principal strain distribution and 2D planar configuration of the Cu/PI composite layer on the square-ring kirigami FSS under biaxial tensile strain were determined by applying the corresponding strain to the ecoflex substrate. To eliminate the influence of boundary effects, equal tensile displacements were applied along the in-plane *x* and *y* directions to a 5 × 5 unit cell structure, focusing on the configuration changes and strain distribution of the central unit cell. Given the significant thickness difference between the Cu/PI composite layer and the ecoflex substrate, shell elements S4R and 3D solid elements C3D8R were used to construct finite element models for the Cu/PI composite layer and the ecoflex substrate, respectively, to improve computational efficiency and accuracy. Linear elastic constitutive relationships were employed for ecoflex, PI, and Cu, with the following elastic moduli *E* and Poisson’s ratios *ν*: *E*_ecoflex_ = 0.06 MPa, *ν*_ecoflex_ = 0.49, *E*_PI_ = 2.5 GPa, *ν*_PI_ = 0.34, *E*_Cu_ = 119 GPa, and *ν*_Cu_ = 0.34. To ensure computational accuracy, mesh size convergence tests were performed.

The free-space EM measurement experiment is described as follows. EM waves are emitted by a transmitting horn antenna to illuminate the sample and are captured by a receiving horn antenna after transmission through the sample. A measurement is conducted prior to sample installation to record the “background signal”, and another measurement is taken after sample installation to obtain the “sample signal”. Coaxial cables collect the EM wave data and transmit it to a vector network analyzer (VNA, R&S ZNB20, ROHDE-SCHWARZ, Munich, Germany). By comparing the energy ratios of EM waves at various frequencies between the two measurements, the transmission coefficient is determined. Horn antennas are mounted on a ground rail to ensure proper focusing of the EM waves and their incidence at the desired angle. To prevent stray EM waves from bypassing the sample and affecting measurement accuracy, pyramidal microwave-absorbing materials are placed around the sample. The sample is stretched under different biaxial strains using a tensile device equipped with 3D-printed clamps, enabling measurement of the transmission coefficient under varying strain conditions.

The EM simulation process is described as follows. For each biaxial strain, ABAQUS is used to simulate and obtain the deformed shapes of the Cu, PI, and ecoflex layers. The deformed mesh elements are then sequentially imported into HyperMesh 2022 (a finite element software for pre- and post-processing) to acquire the corresponding geometric configurations. These configurations under different biaxial strains are subsequently imported into CST for further simulation. EM simulations are performed using a frequency-domain analysis model, where hexahedral elements are used to mesh the square-ring kirigami FSS. For the boundary conditions, a “unit cell” is selected along the *x* and *y* directions, and “open (add space)” is chosen along the *z* direction. The relative permittivity of Ecoflex and PI is 3.4 [44], and Cu is simplified as a perfect electric conductor (PEC).

## Data Availability

Data are unavailable due to privacy.
